# Exploring factors influencing patient activation in Saudi rheumatoid arthritis patients: A Nationwide Cross‐Sectional Survey—Results from the COPARA study

**DOI:** 10.1002/iid3.1101

**Published:** 2023-11-27

**Authors:** Haya M. Almalag, Maha M. Alshehri, Nouf A. Altokhais, Ghada A. Aljanobi, Maha I. El Dessougi, Amal AlHarthi, Maha A. Omair, Suzan M. Attar, Sami M. Bahlas, Abdullah S. Alfurayj, Mansour S. Alazmi, Alhussain M. Asiri, Mohammed M. AlOmair, Lobna I. Al Juffali, Mohammed A. Omair

**Affiliations:** ^1^ Department of Clinical Pharmacy, College of Pharmacy King Saud University Riyadh Saudi Arabia; ^2^ College of Pharmacy King Saud University Riyadh Saudi Arabia; ^3^ Department of Medicine, Rheumatology Unit Johns Hopkins Aramco Healthcare, Dhahran Saudi Arabia Dhahran Saudi Arabia; ^4^ Department of Medicine, Rheumatology Unit Security Forces Hospital Riyadh Saudi Arabia; ^5^ Department of Statistics and Operations Research, College of Science King Saud University Riyadh Saudi Arabia; ^6^ Department of Medicine, Rheumatology Unit King Abdulaziz University Jeddah Saudi Arabia; ^7^ Department of Medicine, Rheumatology Unit Buraidah Central Hospital Buraidah Saudi Arabia; ^8^ Department of Medicine, Rheumatology Unit Prince Mohammed Medical City Sakaka‐Aljouf Saudi Arabia; ^9^ Department of Medicine, Rheumatology Unit Aseer Central Hospital Abha Saudi Arabia; ^10^ Department of Medicine, Rheumatology Unit King Saud University Riyadh Saudi Arabia

**Keywords:** cross‐sectional study, national study, patient activation measures, patient engagement, rheumatoid arthritis

## Abstract

**Objectives:**

To evaluate patient activation in rheumatoid arthritis (RA) patients using patient activation measure 13 (PAM‐13) on a national level in Saudi Arabia.

**Method:**

A national survey was administered across multiple centers in Saudi Arabia. Patient activation was assessed using the PAM‐13. The Compliance Questionnaire for Rheumatology (CQR) and the RA Impact of Disease (RAID) tool were also administered. The data from the survey were analyzed, and the results were stratified based on activation level. All factors affecting patient activation were explored and reported.

**Results:**

A total of 1241 participants were included. Most of the patients were females (85%), the mean age was 47 (±14), and most patients lived in the central region (47%). The mean (±standard deviation) patient activation score was 578.7 (±13.0). Patient activation was affected by multiple factors: demographic characteristics, such as education, with a beta value of 1.11 (95% confidence interval [CI] 0.64 –1.58, *p* < .001). Higher CQR scores were associated with higher activation levels, with a beta value of 2.61 (95% CI 0.80 –4.44, *p* = .005), and higher RAID scores were associated with lower activation levels, with a beta value of 3.13 (95% CI 1.36 –4.91, *p* = .001).

**Conclusions:**

Patient activation was affected by several demographic characteristics and the impact of RA. A higher activation may improve compliance. Future longitudinal studies are required to confirm these findings and should explore the underlying mechanism of these effects.

## Keypoints

- Patient activation was assessed at the national level.
- Patient activity in rheumatoid arthritis is affected by multiple demographics.
- Disease activity is affected by activation.
- Compliance is affected by the level of activation.

## Graphical abstract

Patient activation was assessed at the national level. Patient activity in RA is affected by multiple demographics. Disease activity is affected by activation. Compliance is affected by the level of activation.

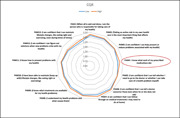

## 1 INTRODUCTION

The Kingdom of Saudi Arabia (KSA) is undergoing an enormous society, economic, and health care‐related transformation in accordance with Saudi Vision 2030.^1^ Empowering people to take the lead in managing their life is one of the fundamental goals of Saudi Vision 2030 transformation. Thus, empowering patients to take the lead in managing their health through activation and engagement is in alignment with the vision goals. The patient is considered the leader of his or her health management and is one of the fundamental advocates in the health care context. The concept of patient activation was introduced by Hibbard et al.^2^ in 2004. It is defined as “an individual's knowledge, skill, and confidence for managing their health and health care.” Patient activation is assessed using a tool that was designed and validated in 2004 and updated and shortened in 2005.^2^, ^3^

Long‐term management of chronic conditions such as rheumatoid arthritis (RA) requires high patient engagement. Although the concept of patient activation was developed recently, it has been explored in multiple countries and in the context of many chronic conditions.^4^, ^5^, ^6^, ^7^ It is important to assess patient activation in patients with chronic conditions because this variable is associated with better disease outcomes.^4^ The level of patient activation among RA patients has been reported to range from 29 to 76.^8^

Patient activation among Saudi patients with RA was recently examined by Al Juffali et al.^8^ Their cross‐sectional survey indicated that Saudi RA patients had a higher level of activation than Western RA patients. However, given the single‐center design and small sample size of their study, the results could not be widely generalized. In addition, Al Juffali et al.^8^ recommended that future research should explore factors affecting patient activation using a nationwide survey. Therefore, the aim of the current research is to assess patient activation using a national, multicenter study. In addition, we aimed to evaluate factors that might affect activation and that could be targeted in educational and awareness campaigns. Furthermore, we aimed to determine the relation of activation with patient‐reported outcomes, such as compliance and the impact of the disease.

## 2 MATERIALS AND METHODS

### 2.1 Study design

The study is the cross‐sectional, nationwide, multicenter survey portion of the COmpliance and Patient Activation in RA (COPARA) study.^9^ The COPARA study has an observational design and is conducted in accordance with the STrengthening of Observational Studies in Epidemiology checklist.^10^

### 2.2 Ethical considerations

The study protocol was approved by the institutional review board of King Saud University (IRB number: E‐19‐4364). Participants provided electronic consent and agreed to participate voluntarily. No identifiable patient data were used, thereby guaranteeing complete patient confidentiality.

### 2.3 Settings

The study included participants with confirmed RA diagnoses across multiple Saudi centers. The centers included King Saud University Medical City (previously known as King Khalid University Hospital), Riyadh; Security Forces Hospital, Riyadh; King Abdulaziz University Hospital, Jeddah; Prince Mohammed Medical City, Al‐jouf; Qatif Central Hospital, Qatif; Buraidah Central Hospital, Buraidah; Aseer Central Hospital, Abha; and the Charitable Association for Rheumatic Diseases database.

### 2.4 Participants

All consecutive adult patients (>18 years) with confirmed RA attending the abovementioned settings were recruited. RA diagnosis was based on the American College of Rheumatology/European League Against Rheumatism classification criteria 2010.^11^ Children and patients who were not taking medication were excluded. Recruitment in each center was performed by one of the research teams between May and June 2021. Participants received a link for an electronic (Google Form) survey via WhatsApp. After electronic consent was obtained, participants completed the predesigned survey.

### 2.5 Variables

The predesigned Google form was used to collect the following variables: demographic characteristics, socioeconomic characteristics, and medication received. In addition, the survey involved two translated and validated surveys. The primary outcome variable was patient activation, which was assessed using the Arabic version of the 13‐item patient activation measure (PAM‐13).^3^ Other outcome variables included disease activity, which was assessed using the translated RA Impact of Disease (RAID) tool,^12^ and compliance, which was assessed using the Arabic version of the Compliance Questionnaire for Rheumatology (A‐CQR).^13^, ^14^

### 2.6 Data source and measurements

The Arabic version of the PAM‐13 was used to evaluate the patients' level of involvement in the self‐management of their disease. Insignia Health granted permission to use the PAM‐13 along with the Arabic version of the survey (license number: 1570198456–1601820856).^3^, ^15^ The original version of the survey included 22 items; subsequently, it was shortened to include 13 items.^2^ The PAM‐13 asks patients to rate the extent to which they agree or disagree with 13 statements on a five‐point Likert scale, with the fifth option indicating “not applicable.” The total score ranges from 0 to 100, with a higher score indicating greater activation. The final score is calculated to divide the patients into four possible levels ranging from 1 to 4, where higher levels indicate higher degrees of activation. The CQR is a short‐form survey that was developed to assess compliance among patients with rheumatic conditions.^13^ It was translated and validated in the Arabic language in 2021.^14^ In the survey, participants are expected to rate the extent to which they agree or disagree with five statements on a 4‐point Likert scale. The RAID tool was originally developed in English and then translated into multiple languages.^12^ The seven‐item tool is used to assess the impact of RA on patients on a scale of 0–10; the higher the score, the worse the impact of RA. The score is then computed to produce a total score.

### 2.7 Bias

Consecutive patients across multiple centers were recruited. Only patients with complete data were included in the analysis.

### 2.8 Study size

The required sample size was calculated based on a proportion of 73 (level 3 and 4 activation), in accordance with Al Juffali et al.^8^ The minimum calculated sample size was 303 participants, which is the number of participants necessary to obtain a confidence level of 95% that the real value is within 5% of the measured value. Although the required minimum sample size was calculated we included almost all of patients with RA that we could recruit at different parts of the kingdom.

### 2.9 Quantitative variables and statistical methods

Data were statistically analyzed using Minitab Statistical Software and the IBM statistical package for social science version 25, Armonk, USA. Continuous variables are reported as the mean and standard deviation. Nonnormally distributed variables are expressed as the median and interquartile range. Categorical variables are expressed as numbers and percentages. Chi‐squared and Fisher's exact tests were used to compare categorical variables. One‐way analysis of variance was used to compare the means of continuous variables across activation levels, and Tukey's test was used for pairwise comparisons. The Kruskal‒Wallis test was used to test differences between groups when appropriate. Multiple linear regression was conducted with PAM‐13 scores as the dependent variable. Factors that were significant in univariate analysis were subsequently entered into the multivariate analysis to predict PAM‐13 scores with resulting beta values, 95% confidence intervals (CIs) and *p* values. Disease impact (assessed by the RAID tool) and compliance (assessed by the CQR) were further evaluated after adjusting for confounding variables.

## 3 RESULTS

**Figure 1:**
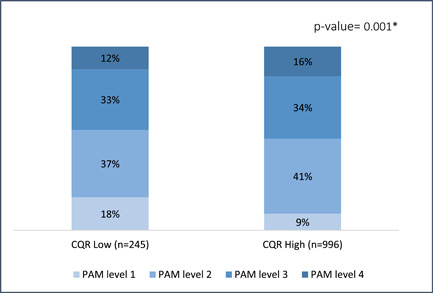
Patient activation measure (PAM‐13) and compliance assessed by the compliance questionnaire for rheumatology (CQR).

**Figure 2:**
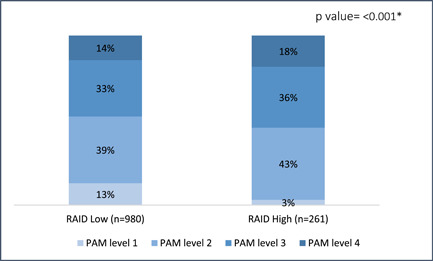
Patient activation measure (PAM‐13) and disease impact by rheumatoid arthritis impact of disease (RAID).

A total of 1241 participants were recruited. The majority of patients were female (85%), and the mean (±standard deviation [SD]) age was 47 (±14). Many patients had a relatively high education, that is, university or diploma (41%), were not working (71%) and had a relatively low income, that is, <3000 SR (53%). The vast majority of patients lived in the central region (47%) and had RA symptoms for more than 10 (±8) years. In addition, participants had mostly an unacceptable impact of disease (79%) and at least one comorbidity (68%). The mean (±SD) PAM score was 58.7 (±13.0). Demographic data were stratified by patient activation level. Participants with level 4 activation had a lower mean age (46 ± 13), were more likely to be female (16%), were more likely to have postgraduate education (30%), were more likely to be working (20%) and were more likely to have an above average income (25%). Participants with level 4 activation mostly lived in the eastern region and had 10 (±8) years of RA symptoms. Participants with level 4 activation were also more likely to report an acceptable level of disease impact on the RAID tool (18%) and more likely to report no or one comorbidity (16% within the same category) compared to two or more comorbidities. There were differences in educational level, employment status, income, region, symptoms of disease, and RAID scores between different levels of activation. These data are shown in Table 1. Univariate analyses of other factors affecting patient activation, such as medication and different comorbidities, are available in Supporting Information: material 1. Only comorbidities or medications with significant differences between activation levels are reported in Table 1. Factors that were significantly different between levels of patient activation were further examined using bar charts. Data on patient activation and compliance (assessed by the CQR) are shown in Figure 1. Data on patient activation and disease impact (assessed by the RAID) are shown in Figure 2.

**Table 1:** Demographic characteristics, rheumatoid arthritis information, and comorbidities divided by PAM level with *p* values.

	PAM level 1	PAM level 2	PAM level 3	PAM level 4	Total	*p* Value
Age, mean (SD)						
Years	45.93 (13.71)	48.05 (13.94)	47.08 (13.77)	45.774 (12.89)	47.14 (13.71)	.165
Gender, *n* (%)						
Male	14 (7.5)	87 (46.8)	60 (32.3)	25 (13.4)	186 (15)	.151
Female	120 (11.4)	410 (38.9)	360 (34.1)	165 (15.6)	1055 (85)	
Educational level, *n* (%)						
No education	21 (13.0)	79 (48.8)	49 (30.3)	13 (8.0)	162 (13.1)	<.001*
Elementary school/read and write	24 (14.1)	78 (45.9)	42 (24.7)	26 (15.3)	170 (13.7)	
Intermediate school	11 (9.9)	50 (45.1)	39 (35.1)	11 (9.9)	111 (8.9)	
High school	29 (12.6)	84 (36.4)	85 (36.8)	33 (14.3)	231 (18.6)	
University or diploma	47 (9.2)	193 (37.6)	182 (35.5)	91 (17.7)	513 (41.3)	
Postgraduate	2 (3.7)	13 (24.1)	23 (42.6)	16 (29.6)	54 (4.4)	
Employment status, *n* (%)						
Not working	107 (12.1)	366 (41.3)	293 (33.1)	120 (13.5)	886 (71.4)	.005*
Working	27 (7.6)	131 (36.9)	127 (35.8)	70 (19.7)	355 (28.6)	
Income, *n* (%)						
<3000 Saudi Riyals	86 (13.1)	289 (43.9)	204 (31)	79 (12)	658 (53.0)	<.001*
3000–10,000 Saudi Riyals	33 (9.4)	136 (38.9)	126 (36)	55 (15.7)	350 (28.2)	
10,000–20,000 Saudi Riyals	14 (7)	60 (30.2)	76 (38.2)	49 (24.6)	199 (16.0)	
>20,000 Saudi Riyals	1 (2.9)	12 (35.3)	14 (41.2)	7 (20.6)	34 (2.7)	
Living status, *n* (%)						
Alone	4 (9.3)	19 (44.2)	16 (37.2)	4 (9.3)	43 (3.5)	.686
With family	130 (10.9)	478 (39.9)	404 (33.7)	186 (15.5)	1198 (96.5)	
Smoking status, *n* (%)						
No	121 (10.8)	451 (40.1)	381 (33.9)	172 (15.3)	1125 (90.7)	.95
Yes	9 (12.3)	29 (39.7)	22 (30.1)	13 (17.8)	73 (5.9)	
Former smoker	4 (9.3)	17 (39.5)	17 (39.5)	5 (11.6)	43 (3.5)	
Region, *n* (%)						
Central region	71 (12.2)	208 (35.8)	225 (38.7)	77 (13.3)	581 (46.8)	<.001*
Western region	18 (6.6)	101 (37.3)	96 (35.4)	56 (20.7)	271 (21.8)	
Eastern region	18 (15)	31 (25.8)	42 (35)	29 (24.2)	120 (9.7)	
Southern region	26 (19)	44 (32.1)	43 (31.4)	24 (17.5)	137 (11.0)	
Northern region	1 (0.8)	113 (85.6)	14 (10.6)	4 (3)	132 (10.6)	
Start of symptoms, mean (SD)						
Years	11.15^a,b^ (8.09)	9.55^b^ (7.07)	11.62^a^ (8.80)	9.69^b^ (7.69)	10.44 (7.95)	<.001*
Diagnosis, mean (SD)						
Years	9.25^a,b^ (7.28)	8.09^b^ (6.83)	9.81^a^ (8.11)	7.90^b^ (7.19)	8.77 (7.43)	.001*
RAID score, median (interquartile range)						
Score	5.59 (3.81–7.36)	4.46 (2.3–5.81)	4.74 (2.22–6.14)	3.95 (1.88–6.48)	4.64 (2.37–6.19)	<.001*
RAID interpretation, *n* (%)						
Unacceptable	126 (12.86)	386 (39.39)	326 (33.27)	142 (14.49)	980 (79.0)	<.001*
Acceptable	8 (3.1)	111 (42.5)	94 (36)	48 (18.4)	261 (21.0)	
CQR, *n* (%)						
Low	44 (18)	90 (37)	81 (33)	30 (12)	246 (20)	<.001*
High	90 (9)	407 (41)	339 (34)	160 (16)	996 (80)	
Rituximab, *n* (%)						
Yes	20 (20.83)	28 (29.17)	34 (35.42)	14 (14.58)	96 (7.7)	.005*
Sulfasalazine, *n* (%)						
Yes	11 (13.41)	23 (28.05)	27 (32.93)	21 (25.61)	82 (6.6)	.020*
Stress, *n* (%)						
Yes	17 (21.5)	20 (25.3)	36 (45.6)	6 (7.6)	79 (6.4)	<.001*
Fibromyalgia, *n* (%)						
Yes	9 (30)	8 (26.7)	9 (30)	4 (13.3)	30 (2.4)	.007*
Comorbidities, *n* (%)						
No or one	85 (10.1)	341 (40.3)	283 (33. 5)	137 (16.2)	846 (68.1)	.406
Two or more	49 (12.4)	156 (39.5)	137 (34.7)	53 (13.4)	395 (31.9)	

*Note*: ab: Means with different letters are significantly different.

Abbreviations: CQR, Compliance Questionnaire for Rheumatology; PAM, patient activation measure; RAID, rheumatoid arthritis impact of disease; SD, standard deviation.

* Significant at a significance level of <.05.

**Figure 3:**
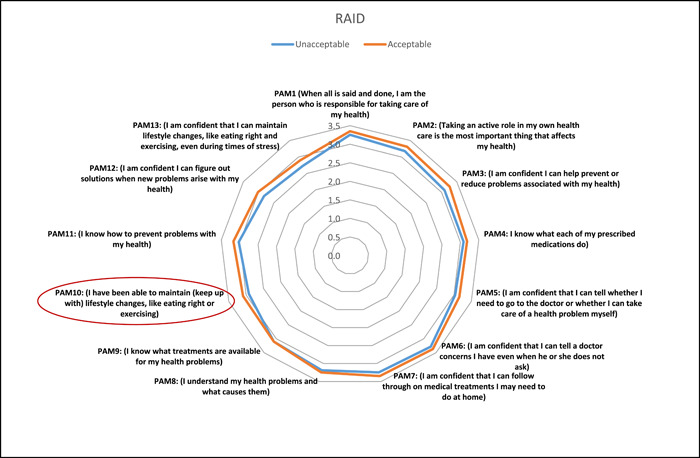
Radar chart for different items of the patient activation measure (PAM‐13) according to rheumatoid arthritis impact of disease (RAID) level with red lines on different items.

**Figure 4:**
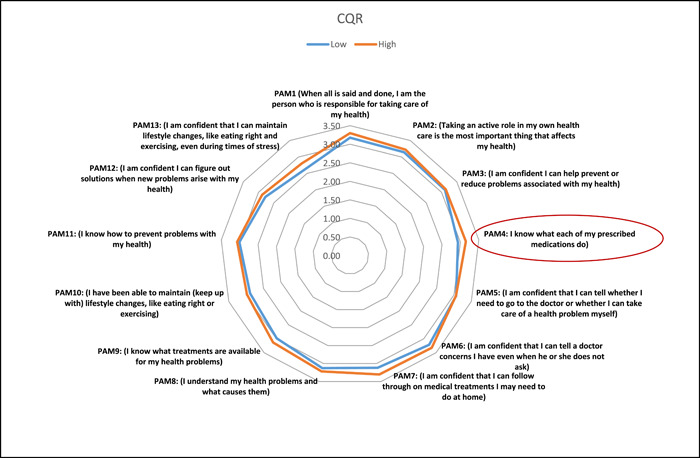
Radar chart for different items of the patient activation measure (PAM‐13) according to compliance level (CQR) with red lines on different items.

Linear regression was used to predict patient activation as a continuous variable. Education level was positively related to activation, with a beta value of 1.11 (95% CI 0.64–1.58, *p* < .001). Employment and income were also related to positive activation, with beta values of 2.17 (95% CI 0.57 –3.77, *p* = .008) and 0.85 (95% CI 0.50 –1.21, *p* < .001), respectively. For residency, the Eastern region seems to predict a lower level of activation, with a beta value of −0.80 (95% CI −1.32 to −0.280, *p* = .003). Some factors related to disease duration from diagnosis and duration from the start of symptoms were significant in the univariate analysis but did not show a significant correlation with PAM‐13 scores. With regard to the impact of disease (assessed using the RAID tool), there was an inverse relationship between RAID scores and activation levels, with a beta value of −0.50 (95% CI −0.78 to −0.22, *p* = .001); people who reported an acceptable level of disease impact on the RAID tool were more likely to have a higher level of activation, with a beta value of 3.13 (95% CI 1.36–4.91, *p* = .001). Compliance, which was assessed by the CQR, was positively related to activation, with a beta value of 2.61 (95% CI 0.80–4.44, *p* = .005). Other factors that were significant contributors to activation in univariate analysis were not shown to be significant predictors in the linear regression model, including rituximab, sulfasalazine, and fibromyalgia. Interestingly, being stressed was inversely correlated with activation, with a beta value of −3.12 (95% CI −6.10 to −0.15, *p* = .039). RAID and CQR scores were further explored using multiple linear regression with adjustment for confounding variables. The associations remained significant, and the relationship with activation was not changed after adjustment. Linear and multiple linear regression values are shown in Table 2. To further explore the associations of the impact of disease and compliance with PAM‐13 scores, we plotted radar charts for PAM‐13 items and RAID and CQR items. It was clear that the item on the RAID tool that had the strongest predictive effect on activation was the ability to maintain lifestyle changes (Figure 3), while the item on the compliance measure with the strongest effect was knowledge of medication (Figure 4).

**Table 2:** Multiple linear regression of factors affecting PAM scores.

Model	Unstandardized coefficients		T	*p* Value	95.0% confidence interval for B	
	B	Std. Error			Lower bound	Upper bound
Educational level	1.108	0.240	4.614	<.001*	0.637	1.580
Employment status	2.167	0.817	2.653	.008*	0.565	3.770
Income	0.852	0.180	4.728	<.001*	0.499	1.206
Region	−0.800	0.265	−3.019	.003*	−1.321	−0.280
Onset of symptoms	−0.019	0.047	−0.414	.679	−0.111	.072
Diagnosed	−0.045	0.050	−0.893	.372	−0.142	.053
RAID score	−0.498	0.143	−3.480	.001*	−0.778	−0.217
RAID acceptable	3.132	0.904	3.465	.001*	1.358	4.906
CQR high	2.622	0.927	2.829	.005*	0.803	4.441
Rituximab	−1.474	1.385	−1.064	.288	−4.191	1.244
Sulfasalazine	2.611	1.488	1.754	.080	−0.309	5.531
Stress	−3.124	1.514	−2.064	.039*	−6.094	−0.154
Fibromyalgia	−3.772	2.408	−1.567	.117	−8.497	.952

**Adjusted for factors significant in univariate analysis (education, employment, income, residency, start of symptoms, years of diagnosis, rituximab, sulfasalazine, stress, and fibromyalgia)**						
RAID acceptable	3.119	0.927	3.366	.001*	1.301	4.937
CQR high	3.195	0.919	3.476	.001*	1.392	4.999

Abbreviations: CQR, Compliance Questionnaire for Rheumatology; PAM, patient activation measure; RAID, rheumatoid arthritis impact of disease.

* Significant at a significance level of <.05.

## 4 DISCUSSION

The study explored patient activation using the PAM‐13 across multiple centers and regions in the KSA. The current study is a continuation of the COPARA project, which aims to explore patient compliance and activation in the RA population. It was confirmed that patient activation was affected by multiple demographic characteristics, such as education, income, and geographical region. In addition, this study found an inverse relation between activation and the impact of disease. The results also highlighted the positive effects of activation on compliance. In a recent systematic review, patient activation in RA was reported.^8^ The review included 19 articles that reported PAM‐13 scores either as a continuous variable or as a categorical variable. The topic was described in general, and there was a great deal of inconsistency between studies that limited the ability to combine results in qualitative synthesis. The reported studies had a limited number of included participants compared to the current work, thus enhancing the importance of the current study findings. In one of the largest multicentre studies, PAM‐13 scores of 1025 patients who were participating in a patient support program related to adalimumab use were determined.^16^ Although the study included many participants, PAM‐13 scores were reported as a continuous variable and not as levels; therefore, it was difficult to categorize patients into different levels of activation. In addition, another large study of 3390 participants involved evaluating activation in patients with chronic illnesses by Blackmore in the United Kingdom; however, not all of the patients had RA.^17^ As a result, the current study could be one of the largest to explore activation among RA patients.

Regarding the global literature, the level of activation reported herein was similar to that of Huang et al.^18^ in Singapore and McBain et al.^19^ in the United Kingdom. The PAM‐13 scores of the Saudi population were lower than those reported by Al Juffali et al.^8^ in a single‐center study. A possible explanation for the relatively high activation was the availability of specialized multidisciplinary clinics at King Saud University and Medical City. To our knowledge, there have been no study reports on the effect of specialized clinics and patient activation. Therefore, this could be an important factor to explore in future studies.

Demographic characteristics related to higher levels of activation are important to consider. Although demographic characteristics are considered unmodifiable factors, understanding areas of strength and weakness of the population is important. Patient activation was positively related to education. This finding was supported by previous literature.^20^ Income and employment were also positively related to activation. Both income and employment findings were not supported by evidence but are factors that could be directly related to higher education. One of the most important factors that inversely affected activation was stress. Zakeri et al.^21^ found that stress was inversely related to activation, and he recommended that health care providers should pay attention to stress when managing the patient as it has an impact on patient‐related outcomes such as quality of life and activation. A factor that we could not explain in detail was differences in activation between regions. These differences may be due to differences in health care settings or health care delivery. It is important to perform a qualitative study to further investigate this finding.

It is important to understand the level of patient activation among patients with chronic illnesses such as RA because active patients have better outcomes.^21^ One of the outcomes reported herein was the impact of disease; it was clear that patients who reported an acceptable level of disease impact had higher levels of activation as the item that most strongly differentiated patients with acceptable and unacceptable disease impacts was adapting to lifestyle changes. This is consistent with the literature as RA patients' outcomes are affected by lifestyle habits.^22^ Another important outcome was the relationship between activation and compliance. A positive relationship was reported in the literature; thus, ensuring that the patient is active is very important.^23^ A very important finding provided by the radar chart was that the most important item that impacted compliance was medication knowledge; thus, focusing on educating patients about their medication could be one of the most important clinical recommendations of this project. To add, the presence of chronic comorbidities was inversely related to activation level.^24^ Our work supports this notion, and suggested theory behind this is that the more comorbidity, the more complex is the therapeutic regimen hence more difficult to engage sufficiently in self‐care.

One strength of this study is that it is the first study to explore factors affecting patient activation using this methodology. In addition, the large number and multicentre nature of this work increase its generalizability. The most important limitations are the cross‐sectional nature of the study and the lack of disease activity measures.

In conclusion, this work is one of the largest studies examining activation in patients with RA. It clearly highlighted demographic characteristics related to activation. It also indicated the importance of activation and its relation to disease outcomes such as disease impact and compliance. Future studies should focus on evaluating patient activation longitudinally and assessing the impact of different interventions.

## AUTHOR CONTRIBUTIONS

All authors contributed significantly to this work and agreed on submission to *Clinical Rheumatology*.

## CONFLICT OF INTEREST STATEMENT

The authors declare no conflict of interest.

## ETHICS STATEMENT

The study protocol was approved by the Institutional Review Board of King Saud University (IRB number: E‐19‐4364). Participants provided electronic consent and agreed to participate voluntarily.

## Supporting information

Supporting information.

Supporting information.

## Data Availability

Data from the project are presented in the main manuscript.
