# Supplementary material for: Exploring factors influencing patient activation in Saudi rheumatoid arthritis patients: A Nationwide Cross‐Sectional Survey—Results from the COPARA study
Source: Immun Inflamm Dis. 2023 Nov 27;11(11):e1101. doi: 10.1002/iid3.1101 (PMC10681035; doi:10.1002/iid3.1101)
Supplement: Supplementary file 1 — Supporting information. [file IID3-11-e1101-s001.docx]

Table 1 comorbidities and PAM level with p value of difference

|  | | **PAM level 1** | **PAM level 2** | **PAM level 3** | **PAM level 4** | **Total** | ***p-value*** |
| --- | --- | --- | --- | --- | --- | --- | --- |
| **Hypertension**, n (%) | Yes | 24 (9.6) | 100 (39.8) | 88 (35.1) | 39 (15.5) | 251 (20.2) | 0.897 |
| **Diabetes**, n (%) | Yes | 27 (12.7) | 93 (43.7) | 61 (28.6) | 32 (15) | 213 (17.2) | 0.290 |
| **Thyroid Diseases**, n (%) | Yes | 21 (10.6) | 83 (41.7) | 63 (31.7) | 32 (16.1) | 199 (16.0) | 0.896 |
| **Osteoporosis**, n (%) | Yes | 26 (16.4) | 45 (28.3) | 66 (41.5) | 22 (13.8) | 159 (12.8) | 0.002* |
| **Increase lipid panel**, n (%) | Yes | 20 (16) | 48 (38.4) | 44 (35.2) | 13 (10.4) | 125 (10.1) | 0.120 |
| **Asthma**, n (%) | Yes | 17 (15.5) | 44 (40) | 32 (29.1) | 17 (15.5) | 110 (8.9) | 0.357 |
| **Stress,** n (%) | Yes | 17 (21.5) | 20 (25.3) | 36 (45.6) | 6 (7.6) | 79 (6.4) | <0.001* |
| **Heart Diseases**, n (%) | Yes | 10 (16.1) | 25 (40.3) | 16 (25.8) | 11 (17.7) | 62 (5.0) | 0.359 |
| **Depression**, n (%) | Yes | 7 (16.3) | 13 (30.2) | 14 (32. 6) | 9 (20.9) | 43 (3.5) | 0.354 |
| **Fibromyalgia**, n (%) | Yes | 9 (30) | 8 (26.7) | 9 (30) | 4 (13.3) | 30 (2.4) | 0.007* |
| **Cancers**, n (%) | Yes | 3 (30) | 2 (20) | 4 (40) | 1 (10) | 10 (0.8) | 0.191 |
| **Viral hepatitis**, n (%) | Yes | 0 (0) | 3 (30) | 3 (30) | 4 (40) | 10 (0.6) | 0.145 |
| **Other**, n (%) | Yes | 66 (10.2) | 269 (41.4) | 222 (34.2) | 93 (14.3) | 650 (52.4) | 0.559 |

Note: *Significant at a significance level of <0.05

Table 2 disease modifying agents and classes of disease modifying agents distributed by PAM level with p value of difference

|  | **PAM level 1** | **PAM level 2** | **PAM level 3** | **PAM level 4** | **Total** | ***p*-value** |
| --- | --- | --- | --- | --- | --- | --- |
| **Individual medication, n (%)** | | | | | | |
| **Methotrexate**, oral tablets or injection | 75 (11.66) | 234 (36.39) | 229 (35.61) | 105 (16.33) | 643 (51.8) | 0.057 |
| **Rituximab**, intravenous injection | 20 (20.83) | 28 (29.17) | 34 (35.42) | 14 (14.58) | 96 (7.7) | **0.005*** |
| **Hydroxychloroquine**, oral tablets | 45 (11.34) | 170 (42.82) | 117 (29.47) | 65 (16.37) | 397 (32.0) | 0.171 |
| **Sulfasalazine**, oral tablets | 11 (13.41) | 23 (28.05) | 27 (32.93) | 21 (25.61) | 82 (6.6) | **0.02*** |
| **Etanercept**, subcutaneous injection | 10 (6.71) | 64 (42.95) | 49 (32.89) | 26 (17.45) | 149 (12.0) | 0.317 |
| **Adalimumab**, subcutaneous injection | 18 (9.14) | 76 (38.58) | 71 (36.04) | 32 (16.24) | 197 (15.9) | 0.755 |
| **Infliximab**, intravenous injection | 5 (21.74) | 8 (34.78) | 8 (34.78) | 2 (8.7) | 23 (1.9) | 0.329 |
| **Abatacept**, subcutaneous or intravenous injection | 5 (15.15) | 12 (36.36) | 12 (36.36) | 4 (12.12) | 33 (2.7) | 0.802 |
| **Anakinra**, subcutaneous injection | 1 (20) | 2 (40) | 0 (0) | 2 (40) | 5 (0.4) | NA |
| **Certolizumab**, subcutaneous injection | 8 (22.86) | 14 (40) | 10 (28.57) | 3 (8.57) | 35 (2.8) | 0.102 |
| **Tocilizumab**, subcutaneous or intravenous injection | 1 (1.75) | 28 (49.12) | 21 (36.84) | 7 (12.28) | 57 (4.6) | 0.099 |
| **Tofacitinib**, oral tablets | 6 (15.38) | 9 (23.08) | 14 (35.9) | 10 (25.64) | 39 (3.1) | 0.088 |
| **Baricitinib**, oral tablets | 1 (9.09) | 4 (36.36) | 3 (27.27) | 3 (27.27) | 11 (0.9) | 0.742 |
| **Upadacitinib**, oral tablets | 2 (15.38) | 4 (30.77) | 3 (23.08) | 4 (30.77) | 13 (1.0) | 0.386 |
| **Leflunomide**, oral tablets | 5 (6.94) | 38 (52.78) | 23 (31.94) | 6 (8.33) | 72 (5.8) | 0.084 |
| **Cortisone**, oral tablets | 40 (12.58) | 116 (36.48) | 118 (37.11) | 44 (13.84) | 318 (25.6) | 0.206 |
| **Classes of disease modifying anti-rheumatic drugs-DMARDs, n (%)** | | | | | | |
| **Monotherapy** | 50 (8.62) | 247 (42.59) | 199 (34.31) | 84 (14.48) | 580 (46.7) | 0.069 |
| **Double therapy** | 54 (12.3) | 155 (35.31) | 156 (35.54) | 74 (16.86) | 439 (35.4) | 0.076 |
| **Triple therapy** | 16 (14.95) | 41 (38.32) | 32 (29.91) | 18 (16.82) | 107 (8.6) | 0.445 |
| **Oral** | 105 (11.27) | 361 (38.73) | 312 (33.48) | 154 (16.52) | 932 (75.1) | 0.106 |
| **Injectable DMARDs** | 67 (12.05) | 211 (37.95) | 196 (35.25) | 82 (14.75) | 556 (44.8) | 0.338 |
| **DMARDs Tumor Necrosis Factor inhibitors (TNF)** | 40 (10.58) | 149 (39.42) | 130 (34.39) | 59 (15.61) | 378 (30.5) | 0.984 |
| **DMARDs non-TNF inhibitors** | 27 (14.84) | 65 (35.71) | 66 (36.26) | 24 (13.19) | 182 (14.7) | 0.157 |
| **Conventional synthetic DMARDs** | 104 (11.09) | 365 (38.91) | 316 (33.69) | 153 (16.31) | 938 (75.6) | 0.253 |
| **Janus kinase inhibitors** | 8 (13.56) | 18 (30.51) | 20 (33.9) | 13 (22.03) | 59 (4.8) | 0.293 |
| **On total oral or injectable medication** | 15 (7.73) | 82 (42.27) | 75 (38.66) | 22 (11.34) | 194 (15.6) | 0.101 |

Note: *Significant at a significance level of <0.05;
